# A combination of GRA3, GRA6 and GRA7 peptides offer a useful tool for serotyping type II and III *Toxoplasma gondii* infections in sheep and pigs

**DOI:** 10.3389/fcimb.2024.1384393

**Published:** 2024-04-24

**Authors:** David Arranz-Solís, Leandro R. Tana, Eduardo Tejerina-de-Uribe, Nadia María López-Ureña, Břetislav Koudela, María E. Francia, Luis Miguel Ortega-Mora, Gema Álvarez-García

**Affiliations:** ^1^ SALUVET group, Faculty of Veterinary Sciences, Animal Health Department, Complutense University of Madrid, Madrid, Spain; ^2^ Laboratory of Apicomplexan Biology, Institut Pasteur de Montevideo, Montevideo, Uruguay; ^3^ Central European Institute of Technology (CEITEC), University of Veterinary Sciences, Brno, Czechia; ^4^ Faculty of Veterinary Medicine, University of Veterinary Sciences, Brno, Czechia; ^5^ Veterinary Research Institute, Brno, Czechia

**Keywords:** *Toxoplasma gondii*, sheep, pig, serotyping, GRA, peptide

## Abstract

The clinical consequences of toxoplasmosis are greatly dependent on the *Toxoplasma gondii* strain causing the infection. To better understand its epidemiology and design appropriate control strategies, it is important to determine the strain present in infected animals. Serotyping methods are based on the detection of antibodies that react against segments of antigenic proteins presenting strain-specific polymorphic variations, offering a cost-effective, sensitive, and non-invasive alternative to genotyping techniques. Herein, we evaluated the applicability of a panel of peptides previously characterized in mice and humans to serotype sheep and pigs. To this end, we used 51 serum samples from experimentally infected ewes (32 type II and 19 type III), 20 sheep samples from naturally infected sheep where the causative strain was genotyped (18 type II and 2 type III), and 40 serum samples from experimentally infected pigs (22 type II and 18 type III). Our ELISA test results showed that a combination of GRA peptide homologous pairs can discriminate infections caused by type II and III strains of *T. gondii* in sheep and pigs. Namely, the GRA3-I/III-43 *vs*. GRA3-II-43, GRA6-I/III-213 *vs.* GRA6-II-214 and GRA6-III-44 *vs.* GRA6-II-44 ratios showed a statistically significant predominance of the respective strain-type peptide in sheep, while in pigs, in addition to these three peptide pairs, GRA7-II-224 *vs.* GRA7-III-224 also showed promising results. Notably, the GRA6-44 pair, which was previously deemed inefficient in mice and humans, showed a high prediction capacity, especially in sheep. By contrast, GRA5-38 peptides failed to correctly predict the strain type in most sheep and pig samples, underpinning the notion that individual standardization is needed for each animal species. Finally, we recommend analyzing for each animal at least 2 samples taken at different time points to confirm the obtained results.

## Introduction

The cyst-forming apicomplexan parasite *Toxoplasma gondii* is the etiologic agent of toxoplasmosis, a worldwide zoonotic disease that can affect virtually all vertebrates, including approximately one third of humans. Toxoplasmosis is considered one of the most important foodborne and waterborne parasitic diseases in the world (Almeria and Dubey, 2021), causing important economic losses in the livestock sector derived from the parasite induced abortions, especially in sheep and goats, and posing also a risk to public health when infected animals are destined for human consumption (Stelzer et al., 2019). Thus, it represents a perfect example of the One Health concept (Aguirre et al., 2019). Among livestock, *T. gondii* infections are usually more prevalent in sheep and pigs than in cattle and poultry (Stelzer et al., 2019), being both animal species extremely relevant for toxoplasmosis epidemiology. Indeed, lamb and pork are considered to be the most relevant meat source of *T. gondii* infection for humans (Belluco et al., 2016, Belluco et al., 2018; Almeria and Dubey, 2021). A range of variables have been proposed as key factors in the development of the different clinical forms of toxoplasmosis, including the strain, life-cycle stage and infective dose of the parasite, the individual host species susceptibility, and the immune status of the host (Mukhopadhyay et al., 2020). Among them, the genetic and phenotypic diversity of *T. gondii* strains constitutes one of the most important causes of variability in the clinical presentation of the disease.

Recent studies based on whole genome sequencing have defined 16 haplogroups assorted into 6 major clades (Su et al., 2012; Lorenzi et al., 2016). *Toxoplasma gondii* strains have a clonal structure, particularly in Europe and North America, where the archetypal lineages type I, II and III comprise the majority of isolated strains (Su et al., 2012). In Europe, type II and, to a lesser extent, type III strains, are the dominating populations, both in domestic and wild environments (Fernández-Escobar et al., 2022), while in North America strains belonging to haplogroup XII also predominate in wild animals (Khan et al., 2011). By contrast, African and Asian *T. gondii* populations remain poorly explored (Chaichan et al., 2017; Galal et al., 2018), and isolates characterized from South America show a greater genetic diversity compared to the archetypal strains, being referred to as non-canonical or atypical strains (Lorenzi et al., 2016). These atypical strains usually exhibit greater virulence and have been associated with more severe clinical symptoms and long-lasting sequelae in humans (Su et al., 2012; Shwab et al., 2016; Hamilton et al., 2019).

Therefore, it is crucial to determine the strain causing infection to better understand the epidemiology of toxoplasmosis, predict its possible clinical consequences and design appropriate control and treatment strategies. To this end, molecular genotyping techniques such as whole genome sequencing (WGS), restriction fragment length polymorphism (RFLP) and microsatellites (MS) have been traditionally applied (Fernández-Escobar et al., 2022), as well as a recently described hi-res next-generation sequencing-based method (Joeres et al., 2024). Although these methods provide high discriminatory power, they possess important inherent limitations. For example, biological samples with enough parasite DNA are required, which usually are only present in tissues with high parasite burdens from clinical or severe cases. In addition, it is necessary to use invasive procedures such as biopsies or lumbar puncture, which are laborious, costly and risky to obtain, and the necessary equipment and procedures required for genotyping are often unaffordable for small laboratories. On the other hand, serotyping methods offer a cost-effective, rapid, sensitive and non-invasive alternative to DNA-dependent techniques, allowing the analysis of subclinical cases (Kong et al., 2003; Sousa et al., 2010; Maksimov et al., 2012a; Arranz-Solís et al., 2019, Arranz-Solís et al., 2021). This technique is based on the detection of antibodies in sera that react against certain segments of antigenic proteins presenting strain-specific polymorphic variations (Dard et al., 2016). Traditionally, these methods have been able to distinguish type II from non-type II strains in humans, mice, cats, sheep, pigs, chicken and turkeys (Kong et al., 2003; Peyron et al., 2006; Sousa et al., 2010; Maksimov et al., 2012b, Maksimov et al., 2013, Maksimov et al., 2018). However, the accuracy of prediction in several of these studies has been low, with many of them relying solely on samples from naturally infected patients or animals where the strain causing infection was unknown. Over the last decade a few studies have tested new peptides using samples from experimental infections performed in mice, cats, rabbits, chicken and turkeys with type I, II and III strains, considerably improving previous results (Maksimov et al., 2013, Maksimov et al., 2018; Arranz-Solís et al., 2019, Arranz-Solís et al., 2021). Of note, by combining different dense granule (GRA) pairs of homologous peptides, Arranz-Solís et al., (2019, 2021) we were able to further differentiate the three clonal lineages from each other and from atypical strains in mouse serum samples. Thanks to this approach, a characteristic and specific fingerprint was obtained for each type of strain, in a similar way to that from genotyping techniques.

Despite all these advancements, several limitations hinder the broad application of serotyping in large-scale epidemiological studies. For example, sequence polymorphisms in antigenic peptides used for serotyping do not always translate into the expected strain-specific reactions (Arranz-Solís et al., 2021). Secondly, it seems that each host species has different reactivity patterns against the same peptides, resulting in a need for individual standardization and characterization of peptides in each host species (McLeod et al., 2012; Maksimov et al., 2013, Maksimov et al., 2018; Arranz-Solís et al., 2021). Finally, there is a critical lack of *bona fide* and reference serum samples, which hampers a thorough characterization of peptides and reliable comparisons among studies (Arranz-Solís et al., 2019, Arranz-Solís et al., 2019; Sousa et al., 2023). In this sense, beyond mice, *T. gondii* experimental infections in other animal species are rare on account of growing ethical concerns, high costs and scarcity of appropriate facilities to allocate and handle livestock (Stelzer et al., 2019). In such experiments, frequently only type II or type III strains, the most prevalent strain types in Europe and North America, are used. In addition, serum samples from natural infections where the causative strain has been isolated and/or genotyped are scarce, and this is especially true for infections caused by strains other than type II or III, such as type I or atypical strains. This hinders serotyping studies attempting to differentiate archetypical and atypical strains or simply describing the specific signature for each strain type in other host species. Indeed, most serotyping studies have focused on analyzing samples from experimentally infected mice or naturally infected humans, and the few studies that have evaluated sera from other animals have used only a very limited number of peptides, samples and/or strain types, with inconsistent results (Kong et al., 2003; Sousa et al., 2010; McLeod et al., 2012; Maksimov et al., 2012a, Maksimov et al., 2013, Maksimov et al., 2018; Arranz-Solís et al., 2019, Arranz-Solís et al., 2021).

Considering the influence of the strains in the epidemiology of toxoplasmosis from both an animal and a public health point of view, in the present study we sought to investigate the usefulness of the panel of peptides previously characterized by Arranz-Solís et al. (2021) in sheep and pigs. To this end, we made use of valuable and well-characterized serum samples obtained from previous experimental infections with different type II and III strains, as well as from natural infections in sheep from which the causing strains were isolated and genotyped. Our results may lay the grounds for future large-scale serotyping studies in these and other animal species.

## Methods

### Experimental design

#### Serum samples

Sheep and pig serum samples used in the present work were obtained in previous studies. A summary of the number of samples of each type and times of collection is provided in **Table 1**. Firstly, serum samples from seropositive pregnant sheep orally infected at day 90 of gestation with 10 or 1000 oocysts of the TgShSp1 (type II, ToxoDB #3), TgShSp16 (type II, ToxoDB #3), TgShSp24 (type III, ToxoDB #2) (Fernández-Escobar et al., 2020) and ME49 (type I, ToxoDB #1) (Sánchez-Sánchez et al., 2019) strains were collected at different times post-infection (pi). Similarly, serum samples from seropositive piglets obtained at 21 and 42 days after being orally infected with 1000 oocysts of the TgShSp1 and TgShSp24 strains (type II and III, ToxoDB #3 and #1, respectively) (Largo-de la Torre et al., 2022), as well as serum samples from prepubertal sows orally infected with 400 oocysts of the CZ-Tiger (type II, ToxoDB #3) and CZ-Šimková (type III, ToxoDB #2) strains (López-Ureña et al., 2023) were used. Further details on each individual sample regarding ID, antibody levels, days post-infection (dpi) or specific uses can be found in **Supplementary File 1**. 

**Table 1:** Summary of the serum samples obtained from experimentally infected sheep and pigs used in the present study.

Animal species and oocyst oral dose	Strain	Genotype (ToxoDB #)	Days post-infection (number of samples)	Reference
Sheep, 10 and 1000 oocysts	TgShSp1	Type II (#3)	14 (2), 21 (8), 27 (1)	(Sánchez-Sánchez et al., 2023; Vallejo et al., 2023)
Sheep, 10 oocysts	TgShSp16	Type II (#3)	19 (3), 34 (4), 50 (8), 68 (4)	(Vallejo et al., 2023)
Sheep, 10 oocysts	TgME49	Type II (#1)	21 (1), 28 (1), 35 (2), 42 (2), 49 (2)	(Sánchez-Sánchez et al., 2019)
Sheep, 10 oocysts	TgShSp24	Type III (#2)	19 (7), 27 (1), 34 (4), 40 (1), 50 (7), 68 (4)	(Vallejo et al., 2023)
Pig, 400 oocysts	CZ-Tiger	Type II (#3)	21 (6), 42 (6)	(López-Ureña et al., 2023)
Pig, 1000 oocysts	TgShSp1	Type II (#3)	21 (5), 28 (1), 42 (5)	(Largo-de la Torre et al., 2022)
Pig, 400 oocysts	CZ-Šimková	Type III (#2)	21 (4), 42 (4)	(López-Ureña et al., 2023)
Pig, 1000 oocysts	TgShSp24	Type III (#2)	21 (5), 42 (5)	(Largo-de la Torre et al., 2022)

For the proof-of-concept assay in field conditions, we used 20 additional samples from naturally infected sheep from which the causative strain was isolated and genotyped: 18 type II and 2 type III strains (Fernández-Escobar et al., 2020) (**Supplementary File 1**). In total, 51 sheep serum samples (32 type II and 19 type III) from 25 experimentally infected ewes, 20 sheep serum samples from natural infections, and 40 serum samples (22 type II and 18 type III) from 20 experimentally infected pigs were analyzed in the present study.

#### Peptide selection

**Figure 1:**
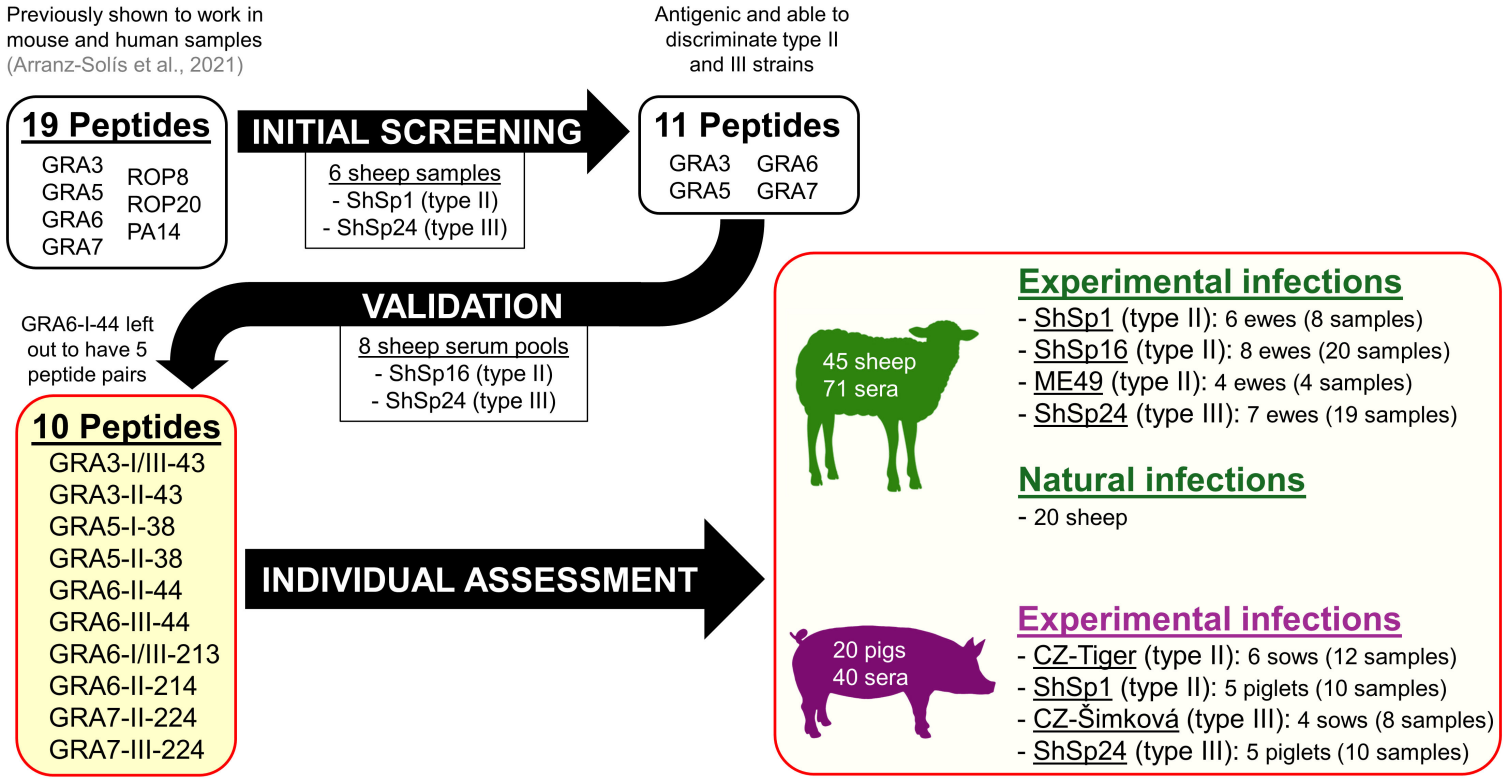
Workflow of the peptide selection and validation process, as well as samples used in the present study.

A panel of 20 peptides previously obtained and characterized in mouse and human serum samples was used (Arranz-Solís et al., 2021). An initial screening was performed to select the most promising peptides to be used in our study, considering their antigenicity and efficiency in discriminating type II and III strains (**Table 2**). To this end, these 20 peptides were assessed against 7 sheep serum samples from pregnant ewes infected with the TgShSp1 and TgShSp24 strains collected at different dpi, so that a representation of different strain types (II and III) and antibody levels (high, mid and low) were included (**Supplementary File 1**, samples indicated with “B” in the column “usage”). A final panel of 11 peptides, all of them derived from GRA proteins, were then validated using 8 serum pools, each obtained after mixing 3 sera from different animals infected with the same strain (either the type II TgShSp16 or the type III TgShSp24) at the same time post-infection (19, 34, 50 or 68 dpi) (**Supplementary File 1**, usage C1). Finally, for the final assessment of all sheep individual samples (**Supplementary File 1**, usages C2 and D), and for logistic reasons (given the high number of samples), we prioritized a panel of 10 GRA peptides (**Table 2**, peptides in red), excluding GRA6-I-44, so that 5 peptide homologous pairs with at least one of the peptides being specific to type II or III strains was available. For the analysis of individual pig samples (**Supplementary File 1**, usage E) the same 10 peptides were used. A workflow chart summarizing the peptide selection process is shown in **Figure 1**.

**Table 2:** List of peptides used in the present study.

Name	Sequence	Initial screening
**GRA3-I/III-43**	ADQP**E**NHQALAEC	Very antigenic and able to differentiate type II and III
**GRA3-II-43**	ADQP**G**NHQALAEC	Very antigenic and able to differentiate type II and III
**GRA5-I-38**	CSEGA**R**G**R**EQ	Antigenic
**GRA5-II-38**	CSEGA**W**G**G**EQ	Antigenic and able to differentiate type II and III
GRA6-I-44	ADS**G**GV**K**QTPC	Antigenic but not needed to differentiate type II and III
**GRA6-II-44**	ADS**G**GV**R**QTPC	Antigenic and able to differentiate type II and III
**GRA6-III-44**	ADS**D**GV**K**QTPC	Antigenic and able to differentiate type II and III
**GRA6-I/III-213**	CLHP**ER**VN**V**FD**Y**	Very antigenic and able to differentiate type II and III
**GRA6-II-214**	CLHP**GS**VN**E**FD**F**	Very antigenic and able to differentiate type II and III
GRA7-I-164*	CLTE**E**QQ**R**GDEP	Lowly antigenic and unable to discriminate strains
GRA7-III-164*	CLTE**Q**QQ**T**GDEP	Lowly antigenic and unable to discriminate strains
**GRA7-II-224**	CVPESG**K**D**G**EDARQ	Lowly antigenic but partially able to discriminate strains
**GRA7-III-224**	CVPESG**E**D**R**EDA	Lowly antigenic but partially able to discriminate strains
GRA7-II-226*	CESG**K**D**G**EDAR	Lowly antigenic but partially able to discriminate strains
GRA7-III-226*	CESG**E**D**R**EDA	Lowly antigenic but partially able to discriminate strains
ROP8-I/III-305*	CSN**T**IKQMK**Q**EV	Lowly antigenic and hardly able to discriminate strains
ROP8-II-305*	CSN**A**IKQMK**E**EV	Lowly antigenic and hardly able to discriminate strains
ROP20-I-331*	CLRKQG**GN**SLLN	Lowly antigenic
ROP20-II/III-331*	CLRKQG**DT**SLLN	Lowly antigenic
PA14-I/II/III-35*	EFRQQHRKTIDGRLC	Lowly antigenic

*Peptides deemed inefficient after a preliminary assessment in sheep samples and thus discarded for further experiments.

Bold type indicates polymorphic sites.

In red: Final peptides selected for the assessment of sheep and pig individual samples (total of 10 peptides).

Readers are referred to Arranz-Solís et al., 2021 for details on peptide origin and gene IDs.

### Sheep and pig enzyme-linked immunosorbent assays (ELISA)

Previous studies from our group have established laboratory developed ELISA protocols for testing sheep and pig serum samples against *T. gondii* infection (Sánchez-Sánchez et al., 2019; Largo-de la Torre et al., 2022; López-Ureña et al., 2023; Vallejo et al., 2023). In the present work, however, we aimed at standardizing sheep and pig ELISAs using the best conditions with a whole tachyzoite soluble antigen, so as to better compare our results to those obtained in the most recent *T. gondii* serotyping work performed with mouse and human samples (Arranz-Solís et al., 2021) while minimizing the background noise.

Thus, the sheep ELISA protocol adopted in the present work was designed by combining the methodology described in previous studies for sheep ELISA using tachyzoite soluble antigen (Castaño et al., 2014) and mouse ELISA using peptides (Arranz-Solís et al., 2021). Briefly, *T. gondii* soluble antigen and each of the selected peptides were used to coat 96-well microtiter plates (10547781, Thermofisher) at 0.15 μg and 1 μg per well, respectively, and incubated overnight at 4°C. Plates were then washed with PBS containing 0.05% Tween 20 (PBS-T) and blocked for 2 h at room temperature with 200 μl blocking solution (5% skim milk in PBS-T). Serum samples were diluted 1:100 in blocking solution, 100 μl added into duplicate wells coated with each of the peptides and the soluble antigen, and subsequently incubated at 37°C for 1 hour. In each plate, the same well-characterized positive and negative control serum samples from previous experiments (Vallejo et al., 2023) were included (**Supplementary File 1**, usage A). Each sample was incubated with the *T. gondii* soluble antigen and all the peptides in parallel within the same plate to obtain results under the same conditions and to better compare the reactivity of the same sample against all the peptides. After washing, 100 μl of a horseradish peroxidase (HRP) conjugate-labeled mouse α-sheep/goat IgG antibody (A9452, Sigma-Aldrich) diluted 1:8,000 in PBS-T were added per well and the plates incubated at 37 °C for 1 hour. Plates were washed thrice as above before the addition of 100 μl per well of ABTS substrate (11684302001, Sigma) and incubated at room temperature in the dark. The optical density (OD) was read at 405 nm (OD_405_) in a plate reader machine (BioTek Synergy H1 Multimode, Agilent) until the positive control incubated with the soluble antigen reached OD values of 1.6-1.8, usually within 25-30 minutes. ODs were recovered using Gen5 version 2.09.1 software (Biotek). Positive/Negative control ratios ranged between 12 and 20 throughout plates.

For pig ELISA, the same protocol described above for sheep was followed with minor modifications. Coating was performed with 0.05 μg and 1 μg per well of soluble antigen and peptides, respectively, while blocking and serum sample dilution preparation and incubation times did not change. As a secondary antibody, the HRP-conjugated protein G (P8170, Sigma-Aldrich) diluted 1:6,000 in PBS-T was used. To develop the enzymatic reaction, ABTS was used as above, and the OD_405_ measured until the positive control incubated with the soluble antigen reached values of 1.0-1.2, usually within 60-90 minutes, after which the reaction was stopped by addition of 100 μl per well of a 0.3N oxalic acid solution. Previously characterized positive and negative serum samples (López-Ureña et al., 2023) were used in each plate, with positive/negative ratios ranging from 11 to 15.

### Relative Index Percentage (RIPC) and peptide ratios

For each sample, OD values obtained with the soluble antigen were relativized by comparison with the positive and negative controls through the Relative Index Percentage (RIPC): (OD_405_ sample – OD_405_ negative control)/(OD_405_ positive control – OD_405_ negative control) × 100. The RIPC values for the *T. gondii* soluble antigen allow the percentage of positivity to be estimated and thus can be considered as an indirect measure of antibody levels for each sample (**Supplementary File 1**).

Similarly, to normalize the reactivity of each sample with the tested peptides, another RIPC value was calculated using the following formula: peptide RIPC = (peptide OD_405_ sample – peptide OD_405_ negative control)/(soluble antigen OD_405_ sample – soluble antigen OD_405_ negative control) × 100. To use a more stringent normalization, constant values for the negative control ODs obtained with the soluble antigen and each of the peptides were used, corresponding to the average of the ODs obtained in all the plates. In sheep, OD values ranged from 0.08 to 0.12 (0.10 average), with a coefficient of variation ranging from 8 to 49% (26% average), while in pigs OD values ranged from 0.08 to 0.10 (0.09 average), with a coefficient of variation ranging from 10 to 36% (18% average).

Finally, and following the same procedure previously described by Arranz-Solís et al. (2021), a quotient, or ratio, between the RIPC values of homologous peptides from different types, for example, GRA6-III-44 *vs.* GRA6-II-44, was calculated for each sample. This value allows for an integrative analysis of the reactivity of each sample against the whole panel of individual peptides, in turn generating a fingerprint that can be used to infer the strain type causing the infection (Arranz-Solís et al., 2021).

### Statistical analysis

In order to compare RIPC values between peptide homologous pairs (for example GRA3-I/III-43 *vs.* GRA3-II-43), unpaired t-tests with Welch’s correction were performed. P-values <0.05 were considered as significant. The agreement between genotyping information and serotyping predictions obtained from each peptide ratio was calculated using the Cohen’s kappa (κ) index with a 95% confident interval. To this end, genotyping was considered 100% specific and sensitive, and type II determination was considered as the diagnosis target for this test, given the overall higher number of type II samples available. Thus, a positive result was considered when a type II prediction was obtained, while type III, I or I/III predictions were considered as negative. In addition, inconclusive predictions were excluded from the analysis. Kappa values were classified as follows: 0.00-0.20: slight agreement, 0.21-0.40: fair agreement, 0.41-0.60: moderate agreement, 0.61-0.80: substantial agreement, and 0.81-1.00: almost perfect agreement. All statistical analysis and graphs were produced using the GraphPad Prism v 8.0.0 software (San Diego, California USA, www.graphpad.com).

## Results

### A panel of 10 GRA peptides differentiates between sheep experimentally infected with type II and type III strains

Based on the results obtained with mouse and human serum samples in the previous work by Arranz-Solís et al. (2021), we decided to initially test 20 peptides using sera with high, mid and low antibody levels from sheep experimentally infected with the TgShSp1 and TgShSp24 strains (type II and III, respectively). After this initial screening, 9 peptides were deemed inefficient: the 4 rhoptry protein (ROP) peptides (ROP8-I/III-305, ROP8-II-305, ROP20-I-331, ROP20-II/III-331), the oocyst-specific PA-14, as well as 4 of the GRA7 peptides (GRA7-I-164, GRA7-III-164, GRA7-II-226 and GRA7-III-226) either failed to discriminate between type II and III strains or presented very low reactivity, and thus were discarded for further use (**Table 2**). By contrast, GRA3-I/III-43, GRA3-II-43, GRA6-I-44, GRA6-II-44, GRA6-III-44, GRA6-I/III-213 and GRA6-II-214 showed promising results and were able to efficiently differentiate between type II and III samples when ratios were calculated (**Supplementary File 2**). In addition to these 7 peptides, we included GRA5-I-38, GRA5-II-38, GRA7-II-224 and GRA7-III-224 in order to have a wider panel of peptides for further experiments. To validate the usefulness of this panel of 11 peptides to predict the strain causing the infection, 8 pools obtained after mixing serum samples from infected animals (ShSp16, type II; and ShSp24, type III) at the same time post-infection were used (**Supplementary File 1**, usage C1). Our results show that the combination of the selected peptides was able to discriminate between all type II *vs.* type III infected sheep samples (**Supplementary File 3**), except for GRA7-III-224 and GRA7-II-224, which showed a ratio that remained below 1 for all samples, although it was much closer to 1 in type III samples. In addition, since the combination of GRA6-II-44 and GRA6-III-44 was enough to discriminate type II and III infections, GRA6-I-44 was not included in subsequent experiments, resulting in a final panel of 10 peptides and 5 peptide ratios for the analysis of individual samples (**Table 2**, in red, and **Figure 1**).

To further corroborate the usefulness of this panel of peptides in individual sheep, 51 sera from ewes experimentally infected with TgShSp1 (Type II, n=8), TgShSp16 (Type II, n=20), ME49 (Type II, n=4) and TgShSp24 (Type III, n=19) were analyzed. Overall, most samples showed expected ratios for their respective strain type, similar to those observed with the sample pools. Nevertheless, because we included samples from 3 different type II strains, we set out to investigate whether different patterns could be found within these groups. TgShSp1 and TgShSp16 showed very similar reactivity patterns, which is not surprising given that both belong to the ToxoDB genotype #3 (henceforth called “Pru-like”, as this genotype was described in the well-known Prugniaud, or Pru, strain). By contrast, results obtained from the ME49 strain (ToxoDB genotype #1) samples showed a general lower reactivity to the set of 10 peptides. Therefore, we considered 3 groups (type II Pru-like, type II ME49-like and type III) to further determine the general reactivity signature, or fingerprint, against the panel of 10 peptides that can be used to predict the strain type causing the infection. To this end, we calculated the average RIPC values and ratios for each of the 3 groups, using both the ratio of RIPC averages and the average of individual ratios. Since no substantial differences were observed between these approaches, we used the ratio calculated from average RIPCs, as it makes more sense mathematically when comparing values below and above 1. The individual OD, RIPC, ratios, averages per group and predictions for each sample are shown in detail in **Supplementary File 4**.

**Figure 2:**
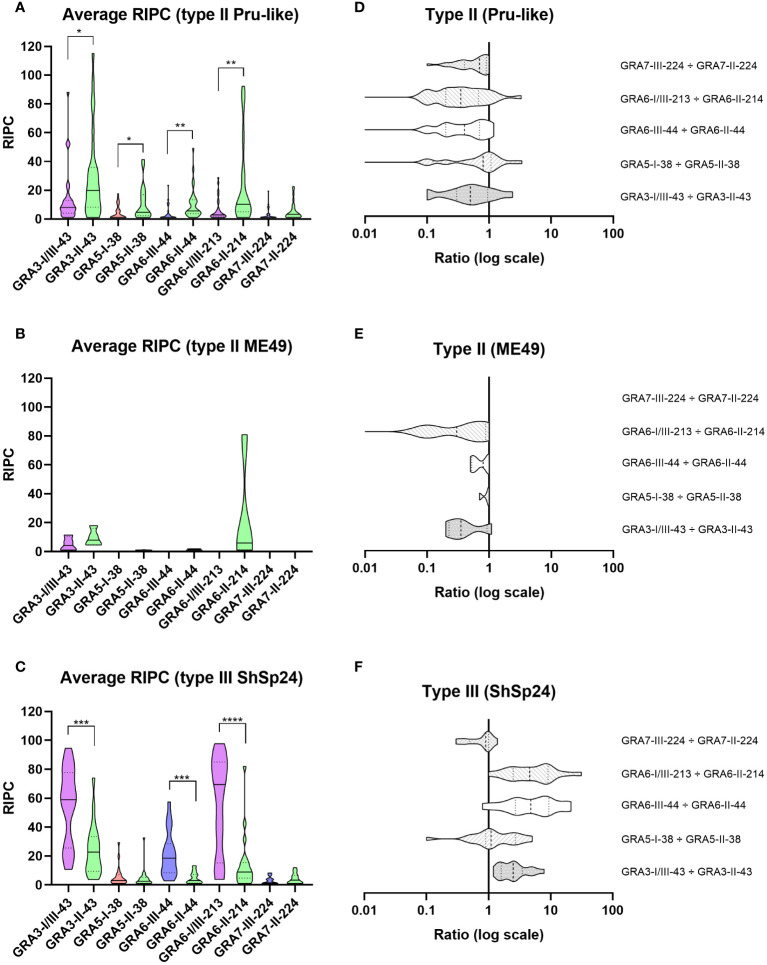
Reactivity signatures in serum samples from sheep experimentally infected with *T. gondii* type II and type III strains when comparing homologous GRA3, GRA5, GRA6 and GRA7 peptides. **(A–C)** Violin plot graphs of the relative index percentage (RIPC) values, showing the mean (solid lines), quartiles (dotted lines) and distribution of data points (dots are not shown to provide a better visualization, individual RIPC values can be found in **Supplementary File 4**) for each group: **(A)** type II Pru-like (ShSp1 and ShSp16, n=28), **(B)** type II ME49-like (ME49, n=4) and **(C)** type III (ShSp24, n=19). Unpaired t-tests with Welch’s correction were performed between homologous peptides pairs. Statistically significant differences are marked with asterisks (**** p<0.0001, *** p<0.001, ** p<0.01, * p<0.05). Type I/III peptides were represented in purple, type I in red, type II in green, and type III in blue. A table with the individual, mean and standard deviation (SD) values is shown in **Supplementary File 4**. **(D–F)** Horizontal violin plot graphs showing the mean (solid lines), quartiles (dotted lines) and distribution of data points (dots are not shown to provide a better visualization) in a logarithmic scale comparing pairs of homologous peptides for each group, resulting from the division of individual RIPC values (**Supplementary File 4**).

**Figure 3:**
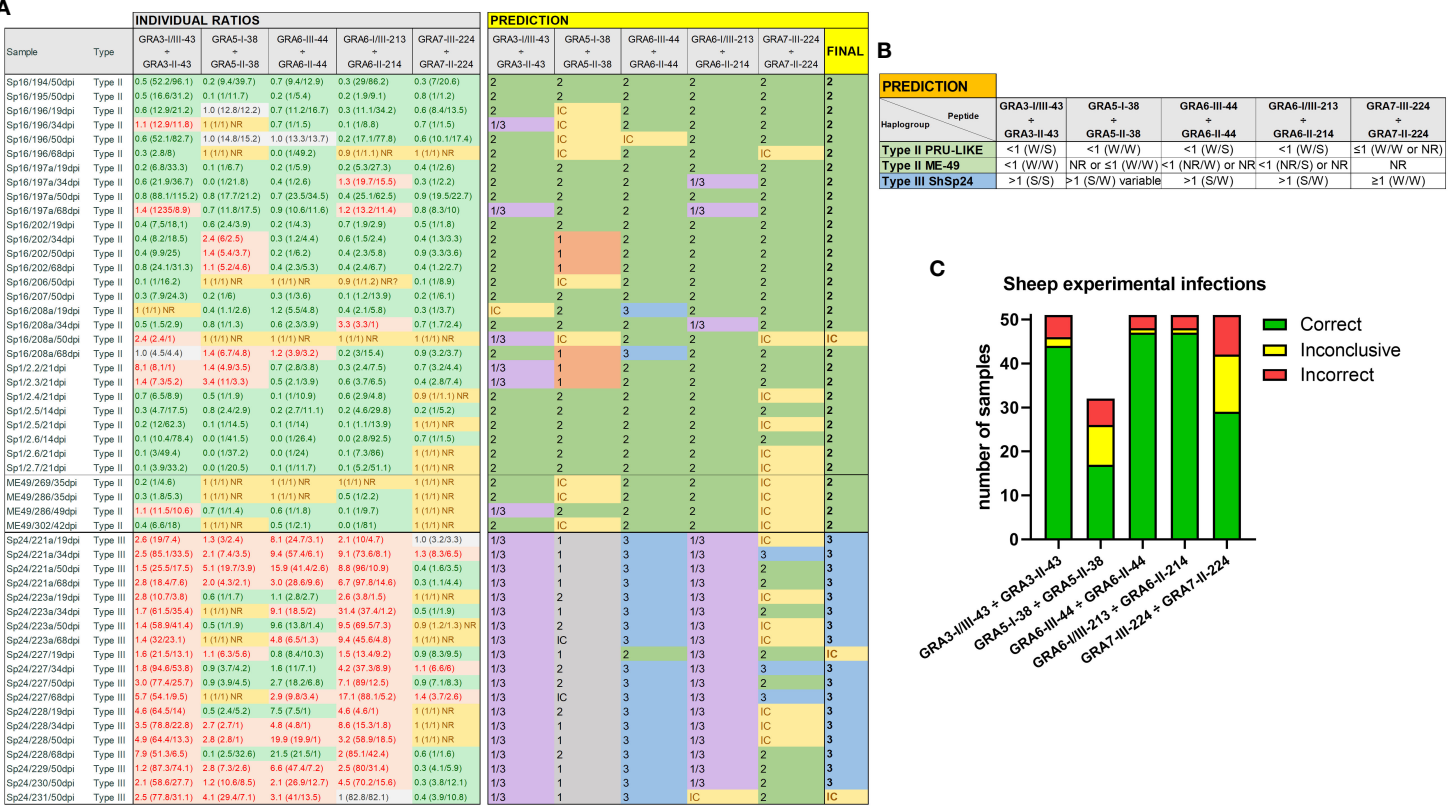
Individual strain type predictions using GRA peptides in sheep serum samples from experimental infections. **(A)** On the left, the individual ratios resulting from dividing the RIPC values for each peptide (in parenthesis) are indicated in red color if values are above 1.05, in green color if values are below 0.95, in grey color if values are between 0.95 and 1.05, and in yellow if both peptides were non-reactive (NR). On the right, individual predictions for each peptide pair are made based on the reference table shown in **(B)**. In the last column on the right, a final prediction was inferred for each sample when at least 3 out of 5 individual predictions were consistent. Otherwise, the sample was marked as inconclusive (IC). Note that for type III (ShSp24) samples, the GRA5-I-38 *vs.* GRA5-II-38 ratio was not considered, as it only differentiates between type I and II strains (in grey). **(C)** Stacked bar graph representing the number of total samples that were predicted correctly (in green), incorrectly (in red), or were inconclusive (in yellow) for each peptide ratio. Since the GRA5-I-38 *vs.* GRA5-II-38 ratio was not considered for type III (ShSp24) samples, the number of total samples for this ratio was lower compared to the others.

After comparing RIPC values between homologous peptide pairs in each strain group, we observed a statistically significant predominance of the respective strain-type peptide for GRA3-I/III-43 *vs.* GRA3-II-43, GRA6-III-44 *vs.* GRA6-II-44 and GRA6-I/III-213 *vs.* GRA6-II-214 in the type II Pru-like and type III ShSp24 groups (**Figures 2A, C**). In addition, the GRA5-I-38 *vs.* GRA5-II-38 comparison also showed a predominance of the second peptide in the type II Pru-like group (**Figure 2A**). By contrast, no significant differences were found between homologous peptides in the type II ME49 group, although only 4 samples were available in this group (**Figure 2B**). Overall, except for GRA7-III-224 *vs*. GRA7-II-224, all ratios exhibited a good prediction level, showing the expected signature for each strain type (**Figures 2D–F**). Considering the individual RIPC values and ratios (**Figure 3A**), we established prediction rules for each strain type group, similar to that described in Arranz-Solís et al. (2021). To this end, both the predominance of the first (>1) and second (<1) peptide, as well as the reactivity (i.e. RIPC intensity) of each individual peptide (non-reactive, weak -W- or strong -S-), were considered to establish the prediction rules. For example, for the GRA3-I/III-43 *vs.* GRA3-II-43 ratio, values >1, being the reactivity of the first peptide strong and the reactivity of the second weak (S/W), would indicate the presence of a type III strain. By contrast, if the ratio is <1 and the reactivity W/S or W/W, then it would indicate that a type II strain is present. This allowed us, when considering all 5 ratios together, to establish a specific fingerprint, or signature, for each strain type group (**Figure 3B**; **Supplementary File 4**), which could be used in turn to infer what strain type caused the infection in further samples.

### GRA3 and GRA6 peptides show the best prediction capacity in sheep sera

In order to validate the predictive potential of this panel of peptides in sheep samples, we used the criteria established in **Figure 3B** to individually analyze each of the experimental infection samples. When considering all ratios together, with at least 3 out of 5 being consistent, the predictions matched the strain in all but three samples (94.1%), which resulted inconclusive: 31/32 type II and 17/19 type III samples (**Figure 3A**). Moreover, there was no variability in the prediction of the strain type depending on the day post-infection (ranging from 19 to 68 dpi), except for two samples that showed inconclusive results in one time point (**Figure 3A**).

Of note, the combination of GRA3-I/III-43 *vs*. GRA3-II-43, GRA6-I/III-213 *vs.* GRA6-II-214 and GRA6-III-44 *vs.* GRA6-II-44 showed the most promising results (**Figure 3C**). Indeed, the Cohen’s kappa index calculated for each individual ratio compared to genotyping information showed a substantial agreement for GRA3-I/III-43 *vs*. GRA3-II-43 (κ=0.76), and an almost perfect agreement for both GRA6-I/III-213 *vs.* GRA6-II-214 (κ=0.87) and GRA6-III-44 *vs.* GRA6-II-44 (κ=0.87). By contrast, GRA5-I-38 *vs.* GRA5-II-38 (κ=0.39) and GRA7-II-224 *vs.* GRA7-III-224 (κ=0.30) showed only a fair agreement. Moreover, when we analyzed the percentage of correct prediction of each peptide pair by group of samples (ShSp1, ShSp16, ME49 and ShSp24), the best results were obtained with GRA6-III-44 *vs.* GRA6-II-44 and GRA6-I/III-213 *vs.* GRA6-II-214, with 88-100% correct predictions, and GRA3-I/III-43 *vs.* GRA3-II-43, with 75-100% correct predictions (**Supplementary File 4**). Conversely, GRA5-I-38 *vs.* GRA5-II-38 showed poor prediction rates, ranging from 25 to 60%, while GRA7-III-224 *vs.* GRA7-II-224 showed to be quite variable in predicting the correct strain type, ranging from 16 to 100%. This latter result can be mainly explained by the low antigenicity detected for most samples, frequently being non-reactive for one or both GRA7 peptides (**Figure 3A**, **Supplementary File 4**). Nevertheless, the only 3 samples exhibiting a GRA7-II-224 *vs.* GRA7-III-224 ratio above 1 belonged to animals infected with the type III strain, while type II samples were either non-reactive or showed ratios below 1.

**Figure 4:**
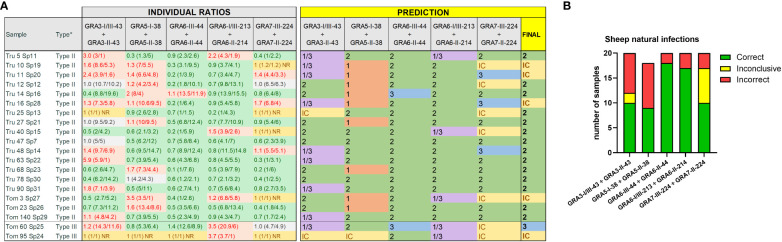
Strain type predictions using GRA peptides in serum samples from naturally infected sheep. **(A)** On the left, individual ratios resulting from dividing the RIPC values for each peptide (in parenthesis) are indicated in red color if values are above 1.05, in green color if values are below 0.95, in grey color if values are between 0.95 and 1.05, and in yellow if both peptides were non-reactive (NR). On the right, individual predictions for each peptide pair are made based on the reference table shown in **Figure 3B**. On the right, in bold, a final prediction was inferred for each sample when at least 3 out of 5 individual predictions were consistent. Otherwise, the sample was marked as inconclusive (IC). *natural infection where the strain type was known based on the genotyping performed on the isolated parasites (Fernández-Escobar et al., 2020). **(B)** Stacked bar graph representing the number of total samples that were predicted correctly (in green), incorrectly (in red) or were inconclusive (in yellow) for each peptide ratio. Since the GRA5-I-38 *vs.* GRA5-II-38 ratio was not considered for type III samples, the number of total samples was lower for this ratio compared to the others.

Once the usefulness of the 10 selected peptides was confirmed in samples from animals under controlled experimental infections, we next sought to determine their usefulness under field conditions. To this end, we used 20 serum samples from naturally infected sheep from which the strain causing the infection was known, as it was previously isolated and genotyped (Fernández-Escobar et al., 2020) (**Supplementary File 1**). In this proof-of-concept assay, we observed a slight decrease in the prediction rate, as some peptides pairs failed to correctly determine the strain type (**Figure 4A**). Nonetheless, the final prediction after considering all ratios together matched the genotype information in 75% of the samples (15/20), being GRA6-II-44 *vs.* GRA6-III-44 the ratio with the highest correct prediction rate (90%, 18/20), followed by GRA6-I/III-213 *vs.* GRA6-II-214 (85%, 17/20) (**Figure 4B**). In contrast to what was observed with the samples from experimentally infected animals, GRA3-I/III-43 *vs.* GRA3-II-43 showed a poor prediction rate, only correctly predicting the strain type in 10 out of 20 samples (50%). Similarly, while in experimental infections a GRA7-III-224 *vs.* GRA7-II-224 ratio above 1 was indicative of the presence of a type III strain, this was not the case in natural infection samples, where only 50% were correctly predicted (10/20). Altogether, based on our results, we recommend including at least the GRA6-213, GRA6-44 and GRA3-43 peptides pairs to identify the strain type in sheep serum samples when at least two of them have coinciding results. In addition, at least 2 samples from different time points should be tested for each animal to confirm the obtained results.

### GRA6 and GRA7 peptides efficiently discriminate *Toxoplasma* type II and III strain infections in pigs

Given the promising results obtained with sheep sera, we set out to investigate the usefulness of the same 10 peptides in pigs. A total of 40 samples from previous experimental infections performed in 20 piglets (Largo-de la Torre et al., 2022) and 20 sows (López-Ureña et al., 2023) were analyzed: 22 serum samples from 11 animals infected with the type II ShSp1 (n=10) and CZ-Tiger (n=12) strains, as well as 18 serum samples from 9 animals infected with the type III ShSp24 (n=10) and CZ- Šimková (n=8) strains (**Table 1**, **Supplementary File 1**).

**Figure 5:**
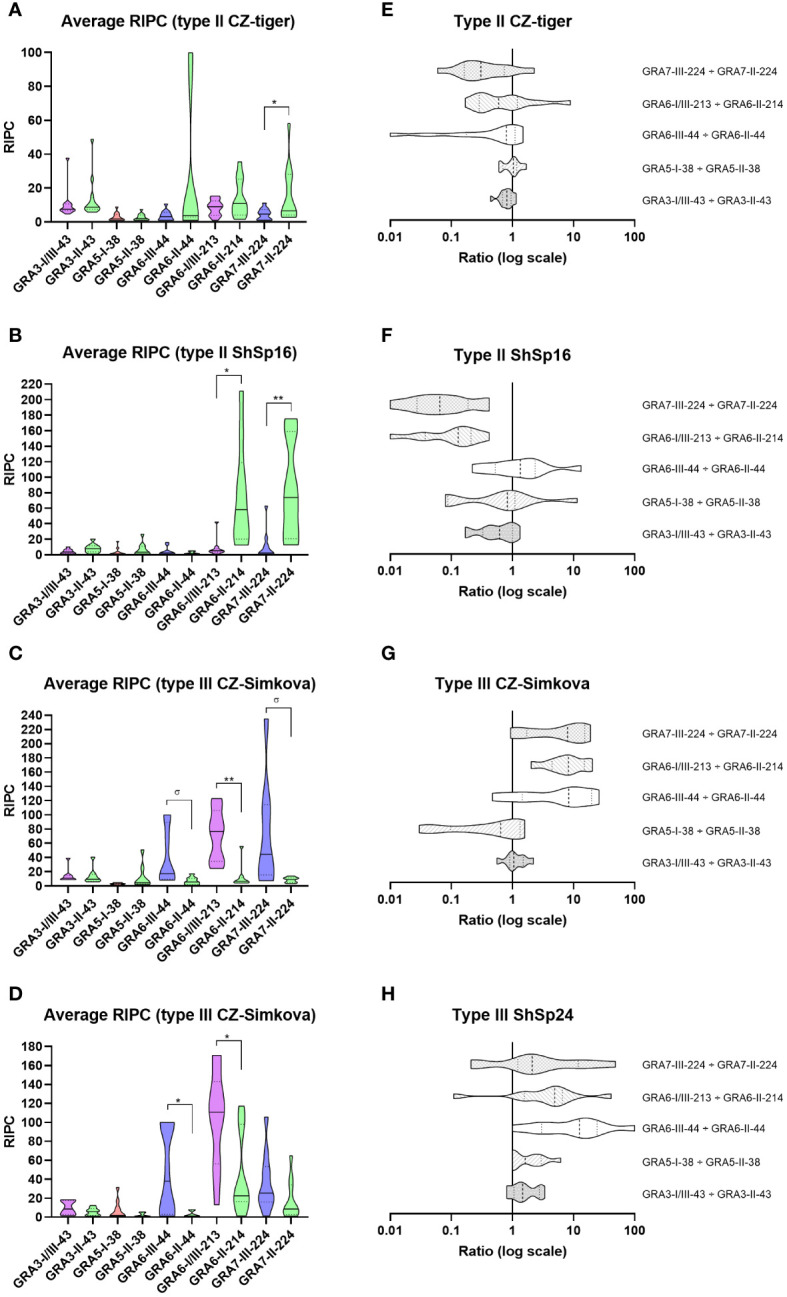
Reactivity signatures in serum samples from pigs experimentally infected with *T. gondii* type II and type III strains when comparing homologous GRA3, GRA5, GRA6 and GRA7 peptides. **(A–D)** Violin plot graphs of the relative index percentage (RIPC) values, showing the mean (solid lines), quartiles (dotted lines) and distribution of data points (dots are not shown to provide a better visualization, individual RIPC values can be found in **Supplementary File 5**) for each group: **(A)** type II CZ-Tiger (n=12), **(B)** type II ShSp16 (n=10), **(C)** type III CZ-Simkova (n=8) and **(D)** type III ShSp24 (n=10). Unpaired t-tests with Welch’s correction were performed between homologous peptides pairs. Statistically significant differences are marked with asterisks (** p<0.01, * p<0.05, σ p=0.05). Type I/III peptides were represented in purple, type I in red, type II in green, and type III in blue. A table with the individual, mean and standard deviation (SD) values is shown in **Supplementary File 5**. **(E–H)** Horizontal violin plot graphs showing the mean (solid lines), quartiles (dotted lines) and distribution of data points (dots are not shown to provide a better visualization) in a logarithmic scale comparing pairs of homologous peptides for each group, resulting from the division of individual RIPC values (**Supplementary File 5**).

Following the same rationale described above for sheep, individual RIPC values were calculated, and a general reactivity signature was established for each strain group by using the ratio of the 5 peptide pairs derived from the respective RIPC averages (**Figure 5**, **Supplementary File 5**). In general, strain groups within the same type (ShSp1 and CZ-Tiger for type II, and ShSp24 and CZ- Šimková for type III) showed similar reactivity patterns, accounting for the fact that they belong to the same ToxoDB genotypes (#3 and #2 for the type II and III strains, respectively), although some peptide ratios showed slight variations within groups (**Figure 5**). When comparing RIPC values between homologous peptide pairs in each strain group, a significant predominance of the respective strain-type peptide was observed for GRA6-III-44 *vs.* GRA6-II-44 in the type III CZ-Simkova group, for GRA6-I/III-213 *vs.* GRA6-II-214 in all groups but type II CZ-tiger, and for GRA7-III-224 *vs.* GRA7-II-224 in both type II groups (**Figures 5A–D**). Furthermore, a trend toward significance (p=0.05) was also observed in the type III CZ-Simkova group for GRA6-III-44 *vs.* GRA6-II-44 and GRA7-III-224 *vs.* GRA7-II-224 comparisons. No significant differences were found between homologous peptides for GRA3-I/III-43 *vs.* GRA3-II-43 and GRA5-I-38 *vs.* GRA5-II-38 comparison in any of the groups. The individual OD, RIPC, ratios, averages per group and predictions for each sample are shown in detail in **Supplementary File 5**.

**Figure 6:**
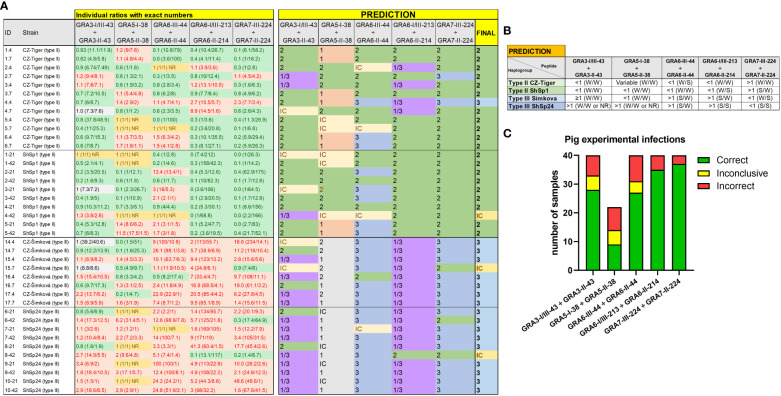
Individual strain type predictions using GRA peptides in experimentally infected pigs. **(A)** On the left, the individual ratios resulting from dividing the RIPC values for each peptide (in parenthesis) are indicated in red color if values are above 1.05, in green color if values are below 0.95, in grey color if values are between 0.95 and 1.05, and in yellow if both peptides were non-reactive (NR). On the right, individual predictions for each peptide pair are made based on the predominance of the first or second peptide in each ratio, considering it as inconclusive (IC) when ratio values ranged between 0.95 and 1.05. In the last column on the right, a final prediction was inferred for each sample when at least 3 out of 5 individual predictions were consistent. Otherwise, the sample was marked as inconclusive (IC). Note that for type III samples, the GRA5-I-38 *vs.* GRA5-II-38 ratio was not considered, as it only differentiates between type I and II strains (in grey). **(B)** A prediction table was made based on the individual RIPC and homologous peptide ratios. Ratios were classified as lower than 1 (<1), higher but close or almost equal to 1 (≥1), higher than 1 (>1), or NR when both peptides in the division had an RIPC value of 1 or were NR. The RIPC reactivity of each peptide is indicated in parenthesis (S, strong; W, weak) and separated by a division mark (/). **(C)** Stacked bar graph representing the number of total samples that were predicted correctly (in green), incorrectly (in red) or were inconclusive (in yellow) for each peptide ratio. Since the GRA5-I-38 *vs.* GRA5-II-38 ratio was not considered for type III samples, the number of total samples was lower for this ratio compared to the others.

The Cohen’s kappa index calculated for each individual ratio compared to genotyping information showed a substantial agreement for GRA6-I/III-213 *vs.* GRA6-II-214 (κ=0.75) and GRA7-II-224 *vs.* GRA7-III-224 (κ=0.75), a moderate agreement for GRA3-I/III-43 *vs*. GRA3-II-43 (κ=0.59), and GRA6-III-44 *vs.* GRA6-II-44 (κ=0.51), and only a slight agreement for GRA5-I-38 *vs.* GRA5-II-38 (κ=0.17). When considering all the ratios together, with at least 3 out of 5 being consistent, the strain type could be correctly inferred in 90% of the samples (36/40), being similar for all 4 groups (88-92%) and with only 1 sample incorrectly predicted (or inconclusive) in each (**Figures 6A, B**). The best prediction rate in pigs was observed with the GRA7-III-224 *vs.* GRA7-II-224 (93% overall correct prediction in 37/40 samples) and GRA6-I/III-213 *vs.* GRA6-II-214 ratios (87% overall correct prediction in 35/40 samples) (**Figure 6C**). Nevertheless, the GRA6 ratio showed a better prediction rate in the ShSp1-infected animals compared to the CZ-Tiger samples (100% *vs.* 67% correct predictions). Likewise, the GRA7 ratio was slightly better at predicting in the ShSp1 compared to CZ-Tiger type II groups (100% *vs.* 83%). Interestingly, the GRA6-III-44 *vs.* GRA6-II-44 ratio that showed very good results in sheep was not as accurate in predicting the strain type in pig samples (67%, 27/40), especially those from the type II genotype, with only 40-58% correct predictions (4/10 and 7/12). Nonetheless, this ratio correctly predicted 16/18 type III samples, being similar in both ShSp24 and CZ-Šimková groups (88-90%). The GRA3-I/III-43 *vs.* GRA3-II-43 ratio showed a variable prediction rate, ranging from 50 to 80% in the different groups (70% overall correct prediction in 28/40 samples), while the GRA5-I-38 *vs.* GRA5-II-38 ratio showed the least accurate prediction rate, with only 33-50% in the type II groups. However, it should be taken into account that only type II samples were considered for this calculation, as the GRA5-III-38 peptide was not available and thus type III strains cannot be correctly inferred with this ratio. In addition, the low prediction rate of the GRA5-I-38 *vs.* GRA5-II-38 can also be explained by the general low reactivity of the samples to these two peptides, which had an average RIPC value of 4 and 6, with 15/40 and 11/40 being non-reactive, respectively (**Supplementary File 5**).

Finally, it is worth mentioning that although there was little variability in the reactivity pattern between samples from the same animal collected at different time points (21 or 42 dpi), 4 samples, 1 from each group, showed different reactivity against several peptides. These samples had low RIPC values; hence it is possible that the reactivity against peptides might not be reliable when *T. gondii* antibody levels are low. Altogether, based on our results, we recommend including GRA7-224, GRA6-213 and either (ideally both) GRA3-43 or GRA6-44 peptide pairs to identify the strain type causing the infection in pig serum samples when at least two of them (or three if 4 peptide pairs are included) have coinciding results. Moreover, for each animal at least 2 samples from different time points should be tested to confirm the reactivity patterns.

## Discussion

Serotyping can play an important role in determining the most prevalent *T. gondii* strains in abortion outbreaks, especially in small ruminants, or in animals with meat products destined for human consumption. Therefore, and considering their relevance for the epidemiology of toxoplasmosis, in the present study we aimed at investigating the usefulness of a panel of peptides previously shown to predict the strain type in mouse and human serum samples, now in domestic sheep and pigs (Arranz-Solís et al., 2021). To this end, we used serum samples from both experimental and natural infections where the causative strain was known, employed a wide panel of peptides and performed a peptide ratio strategy to compare homologous versions, which has been shown to mitigate the inherent individual variability in the reactivity of peptides (Arranz-Solís et al., 2021). Our promising results allowed us to suggest practical recommendations for future large-scale serotyping assays in these species that can be used to predict the strain type causing the infection.

Apart from humans, *T. gondii* serotyping has been previously investigated in other animals, including mice (Kong et al., 2003; Arranz-Solís et al., 2019, Arranz-Solís et al., 2021), cats (Maksimov et al., 2013), chickens (Sousa et al., 2010; Maksimov et al., 2018) and turkeys (Maksimov et al., 2018). To our knowledge, the only attempt in pigs and sheep was performed in 2010 by Sousa et al. In this work, a panel of 11 chicken and 15 pig serum samples from type II and III natural infections where the causative strain was isolated (Dubey et al., 2006; Sousa et al., 2006), as well as field samples from 35 chickens, 29 pigs and 50 sheep for which the strain causing the infection was unknown, were assessed with a panel of 4 GRA6 and GRA7 recombinant peptides consisting of three repetitions of selected polymorphic sequences (Sousa et al., 2008, Sousa et al., 2009). The results obtained with these peptides allowed room for significant improvement as less than 15% of the pig serotypes matched the genotype of the strain, with most of the samples being non-reactive. However, in this work only 4 peptides were used and only the individual OD values were considered for determining the serotype (Sousa et al., 2010). A good serotyping peptide should be reactive enough and have homologous versions for one or more different strains, allowing to compare their reactivity rather than considering each one independently. By relativizing the OD values with that from a whole tachyzoite lysate antigen, a RIPC ratio can be obtained and so determine the predominant peptide in each pair (Arranz-Solís et al., 2021). Based on this criterion, in our study the best peptide pairs to discriminate infections caused by different strains in sheep and pigs were derived from GRA6-213 and GRA6-44 peptides, together with GRA3-38 in sheep and GRA7-224 in pigs.

Although many *T. gondii* proteins have been used in the serodiagnosis of the disease, only a few have been tested for serotyping purposes. In total, to our knowledge, peptides derived from 62 different *T. gondii* proteins have been tested up to date for serotyping (details can be found in Arranz-Solís et al., 2019). Among them, polymorphic regions from GRA, ROP, SRS and other diverse proteins such as OWP, Toxofilin and yet-to-be-described proteins (currently annotated as hypothetical proteins) have been assessed. However, GRA-derived peptides have been shown to provide the most promising results in previous studies where peptides from GRA, MIC, SRS and ROP were evaluated (Kong et al., 2003; Maksimov et al., 2013; Arranz-Solís et al., 2021). Hence, it does not come as a surprise that in our initial assessment in sheep samples of the peptides previously characterized in mice and human sera (Arranz-Solís et al., 2021) the best peptides were derived from GRA3, 5, 6 and 7 proteins. After comparing homologous peptides, the GRA6-II-44 *vs.* GRA6-III-44 ratio was very effective in differentiating infections caused by type II and III strains (especially in sheep), in contrast to what was observed in mice (not antigenic) and humans (not efficient) (Arranz-Solís et al., 2021), confirming that each species might have different reactivity patterns against the same peptides. The GRA3-I/III-43 *vs.* GRA3-II-43 and GRA6-I/III-213 *vs.* GRA6-II-214 ratios resulted highly specific in detecting type II infections in sheep, as all the samples with ratios below 1 belonged exclusively to this group, while reactions above 1 were present in type III infections but some also appeared in type II infections. By contrast, GRA7-III-224 *vs.* GRA7-II-224 ratio showed poor predictive abilities in sheep, especially for type III strains, possibly due to the low reactivity of these 2 peptides with most samples, a fact that was previously reported in mice and human samples (Arranz-Solís et al., 2021). By contrast, this GRA7 ratio was very efficient in predicting the strain type in pig infections. Regardless of the individual reactivity of each peptide, with the combination of the 5 GRA ratios an accurate prediction was achieved in 94% and 90% of the experimentally infected sheep and pig samples, respectively, which confirms that with this strategy the relevance of the individual variability in each animal and sample can be attenuated, as it considers the global vision of the different ratios between homologous versions of peptides.

To evaluate the possible variability depending on the time after infection, we compared the reactivity in several samples from the same animal collected at different time points, ranging from 2 to 9 weeks pi in sheep, and between 3 and 6 weeks pi in pigs. Except for a few samples, particularly in pigs, no substantial differences were found in the global prediction when considering all peptide ratios. However, variations in the reactivity of individual peptides were detected for some animals. This suggests that the time after infection, perhaps related to the antibody levels, could influence the reactivity of these peptides producing a slightly different pattern that in turn may affect the ratio of peptide pairs. Maksimov et al. (2018) reported a gradual reduction in the reactivity of several peptides in experimentally infected turkeys and chickens over time until 9 weeks pi. This pattern was not reproduced in our study. These differences might be explained by the individual variability of the host species regarding antigen recognition, or the intrinsic differences among peptides used in each study. Nonetheless, in our study, and despite the individual differences, the global strain type prediction when considering the 5 peptide pair ratios in the same animal at different time points was not affected, highlighting the importance of using a wide range of homologous peptide pairs for this purpose. Furthermore, since all the sheep and pig serum samples used in our study were obtained after oral infections with oocysts, we could not study the influence of the route of infection or parasite stages in the reactivity and prediction abilities of the tested peptides. As for the influence of the dose of infection, all of our sheep samples belonged to animals infected with 10 oocysts (Sánchez-Sánchez et al., 2019; Vallejo et al., 2023), except for a few that were infected with 1000 oocysts of the TgShSp1 strain (Sánchez-Sánchez et al., 2023). Because the number of the latter samples was low (8), we could not draw any conclusions; however, there did not seem to be a difference in the peptides reactivity when comparing both infection doses. This fact was also observed in the study mentioned above in turkey and chicken samples (Maksimov et al., 2018). In pigs, serum samples were obtained from piglets infected with 1000 or sows infected with 400 oocysts, belonging to different strains. In general, although slightly better prediction rates were observed for piglets infected with 1000 oocysts, no significant differences were observed in the reactivity pattern between these groups. However, since different doses were not used within the same type of animals at the same age or under the same conditions, it is difficult to draw conclusions from this fact. Indeed, it is possible that the higher prediction rate observed in piglets was rather a consequence of their younger age, when a more controlled immunological status would be expected in comparison to adult pigs, which are more likely to have been exposed to other antigens that might elicit unspecific antibodies that react against the peptides.

When we took a closer look at the results obtained with each of the 3 different type II strain sheep serum samples, two different patterns, corresponding to each of the ToxoDB genotype numbers, were observed. Namely, the TgShSp1 and TgShSp16 strains belonging to the ToxoDB #3 genotype (Pru-like) showed very similar patterns, while the ME49 ToxoDB #1 genotype elicited a slightly different response, with general lower reactivity levels. Although Pru and ME49 share the same amino acid sequence in all the segments studied from GRA3, GRA5, GRA6 and GRA7 proteins (Arranz-Solís et al., 2021), the detected reactivity was not identical. It is therefore possible that the presence of immunogenic epitopes in other segments of the proteins might elicit different reactivity in strains that share the same sequence in the epitopes where the peptides are located (Arranz-Solís et al., 2019). However, because we only had 4 ME49 samples, this result needs to be confirmed in future analysis using a larger number of samples and a higher variety of type II strains. In pigs, on the other hand, no significant differences were observed when comparing the two different type II and two different type III strains beyond a slightly better prediction rate for GRA6-I/III-213 *vs.* GRA6-II-214 and GRA3-I/III-43 *vs.* GRA3-II-43 ratios for the ShSp1 type II and the ShSp24 type III infected piglets, respectively. Since the ShSp1 and CZ-Tiger belong to the same ToxoDB genotype #3, and the ShSp24 and CZ-Šimková belong to the same ToxoDB genotype #2 (López-Ureña et al., 2023), similar reactivity patterns were expected. Nonetheless, as stated above, it is possible that these slight differences in prediction rates can be explained by the type of animal infected rather than the isolate itself, as piglets, which showed the better prediction rates, were infected with the ShSp1 and ShSp24 strains (Largo-de la Torre et al., 2022).

Although not as high as experimental infections, the accuracy rate in predicting the strain type in sheep samples from natural infections caused by type II and III strains showed moderate levels (75%), with the GRA6-II-44 *vs.* GRA6-III-44 and GRA3-I/III-43 *vs.* GRA3-II-43 ratios being the most and the least reliable ones, respectively. Samples obtained from natural infections possess inherent limitations, as there exist uncontrolled parameters that might influence the reactivity against peptides, such as the immunological status, infection dose, time post-infection at sampling, cross-reactivity with other pathogens or multiple current or past infections with other *T. gondii* strains, among others. Hence, it is expected that these samples are challenging to assess, especially considering the absence of sera obtained from sheep infected with type I, or even atypical, strains, and the scarcity of sera obtained from the more common type II and III infections where the strain was isolated and/or genotyped (Sousa et al., 2010; Fernández-Escobar et al., 2022). Despite these limitations, and even though in our study only 18 type II and 2 type III samples were available, reactivity profiles could be determined, which would in turn be useful to perform future comparisons with other samples and infer whether those infections are caused by type II, III or other strains showing matching or different reactivity patterns.

In pigs, although no samples from naturally infected animals were studied, our serotyping results from experimental infections with 4 different type II or III strains looked promising, especially the combination of GRA6-44, GRA6-213/214 and GRA7-224 peptides, which showed to be very efficient in differentiating infections caused by type II and III strains, improving the single previous attempt performed by Sousa et al. (2010). Contrary to that observed in sheep samples, the GRA7-III-224 *vs.* GRA7-II-224 ratio showed good results and correctly predicted the strain in 35/40 samples. By contrast, pig samples were low reactive against GRA5-II-38, showing a poor prediction accuracy. In addition, since we used the peptides previously selected in sheep for the pig sera assessment, it is possible that some of the peptides we ruled out in sheep might have worked in pigs and could be worth including in future assays in these and other animals. For example, despite being lowly antigenic, ROP8-I/III(II)-305 peptides could be an interesting option, as our initial assessment revealed ratios that were low but correct in 3/4 sheep samples. Similarly, as mentioned above, GRA6-44 peptides showed much better results in sheep and pigs compared to mice and humans (Arranz-Solís et al., 2021), supporting the notion that peptides that do not work in some animal species might in others and vice-versa. It is therefore crucial to refine and standardize serotyping trials for each animal species, as there are inherent differences and reactivity patterns that might not match previously described ones. Moreover, our results highlight the need for including further samples from animals infected with other strains, including type I and other less common but more clinically and epidemiologically relevant atypical strains, so as to identify potential strain-specific signatures that might allow to define patterns in samples from field conditions.

Finally, two of the GRA5 and GRA6 epitopes in our peptides have a single different version for each of the archetypal strains, I, II and III, in contrast to the other peptides that have biallelic variations, usually I/III and II. Regarding GRA6-44, we only included in the final panel of peptides the type II and III versions, as only serum samples from animals infected with type II and III strains were available. Given the promising results obtained with this peptide ratio, it is tempting to speculate that samples from animals infected with type I strains would show a higher reactivity with the GRA6-I-44 version when compared to the type II and III versions. Thus, in future experiments it might be worth including all the possible peptide versions to compare the reactivity patterns obtained here for type II and III infections. As for the GRA5-38 peptides, only the I and II version were used, as the type III version could not be obtained after several attempts in the past where the lyophilized peptide could not be resuspended (Arranz-Solís et al., 2021). If new peptides batches are ordered in the future, new solvents, such as urea, could be tried to resuspend this peptide and compare its reactivity in sheep, pig, or other species serum samples.

Altogether, our findings support the results previously described by Arranz-Solís et al. (2021) regarding the usefulness of GRA peptides in the serotyping of infections caused by *T. gondii*. We have significantly advanced in the serotyping of two of the most important animal species for *T. gondii* infection epidemiology, sheep and pigs, showing a promising approach that might be useful for future epidemiological studies and to apply a similar strategy in other relevant animal species. In this sense, it is crucial to initially validate the reactivity and usefulness of peptides in each animal species, so as to select the most antigenic and strain-specific ones to be used. A practical recommendation for future serotyping epidemiological studies in sheep and pigs is to include at least GRA6-213, GRA6-44 and GRA3-43 peptide pairs for the former and GRA7-224, GRA6-213 and GRA6-44 for the latter, as well as testing at least 2 samples from the same animal to confirm the obtained results. Finally, further investigation could be conducted to assess the specificity of these peptides against samples from animals infected with other related apicomplexan parasites, such as *Neospora caninum* or *Sarcocystis* spp., or even other common pathogens in sheep and/or pigs, to investigate the possible cross-reactions that might interfere with the interpretation of the *T. gondii* strain type prediction.

## Data availability statement

The original contributions presented in the study are included in the article/**Supplementary Material**. Further inquiries can be directed to the corresponding author.

## Ethics statement

The animal studies where these serum samples were collected were approved by Local and National Animal Welfare committees following proceedings described in EU legislation (Council Directive 2010/63/EU). These previous studies were conducted in accordance with the local legislation and institutional requirements under the following protocol numbers: 416-2016 and 1063/2021 for sheep, and PP 55/2016 and PROEX 293.7/20 for pigs.

## Author contributions

DA-S: Conceptualization, Data curation, Formal analysis, Funding acquisition, Investigation, Methodology, Project administration, Resources, Supervision, Validation, Writing – original draft, Writing – review & editing. LT: Investigation, Methodology, Writing – review & editing. ET: Investigation, Methodology, Writing – review & editing. NL-U: Investigation, Methodology, Writing – review & editing. BK: Resources, Writing – review & editing. MF: Supervision, Writing – review & editing. LO-M: Conceptualization, Funding acquisition, Resources, Supervision, Validation, Writing – review & editing. GÁ-G: Conceptualization, Funding acquisition, Resources, Supervision, Validation, Writing – review & editing.

## Supplementary material

Supplementary File 1 Sheep and pig serum samples used in the present study.

Supplementary File 2 RIPC and ratio values obtained in the initial screening performed in sheep serum samples.

Supplementary File 3 RIPC and ratio values obtained in the validation of the 11 selected peptides with pools of sheep serum samples.

Supplementary File 4 Individual OD, RIPC, ratios, averages per group and prediction rates in the assessment of each sheep serum sample.

Supplementary File 5 Individual OD, RIPC, ratios, averages per group and prediction rates in the assessment of each pig serum sample.

## References

[B1] Aguirre A. A. Longcore T. Barbieri M. Dabritz H. Hill D. Klein P. N.. (2019). The one health approach to toxoplasmosis: epidemiology, control, and prevention strategies. Ecohealth 16, 378–390. doi: 10.1007/s10393-019-01405-7 30945159 PMC6682582

[B2] Almeria S. Dubey J. P. (2021). Foodborne transmission of *Toxoplasma gondii* infection in the last decade. An overview. Res. Veterinary Sci. 135, 371–385. doi: 10.1016/j.rvsc.2020.10.019 33148402

[B3] Arranz-Solís D. Carvalheiro C. G. Zhang E. R. Grigg M. E. Saeij J. P. J. (2021). *Toxoplasma* GRA peptide-specific serologic fingerprints discriminate among major strains causing toxoplasmosis. Front. Cell Infect. Microbiol. 11. doi: 10.3389/fcimb.2021.621738 PMC7935526 33680990

[B4] Arranz-Solís D. Cordeiro C. Young L. H. Dardé M. L. Commodaro A. G. Grigg M. E.. (2019). Serotyping of *Toxoplasma gondii* infection using peptide membrane arrays. Front. Cell Infect. Microbiol. 9. doi: 10.3389/fcimb.2019.00408 PMC6895565 31850240

[B5] Belluco S. Mancin M. Conficoni D. Simonato G. Pietrobelli M. Ricci A. (2016). Investigating the determinants of *Toxoplasma gondii* prevalence in meat: A systematic review and meta-regression. PloS One 11, e0153856. doi: 10.1371/journal.pone.0153856 27082633 PMC4833317

[B6] Belluco S. Simonato G. Mancin M. Pietrobelli M. Ricci A. (2018). *Toxoplasma gondii* infection and food consumption: A systematic review and meta-analysis of case-controlled studies. Crit. Rev. Food Sci. Nutr. 58, 3085–3096. doi: 10.1080/10408398.2017.1352563 29020460

[B7] Castaño P. Fuertes M. Ferre I. Fernández M. Ferreras M. del C.. (2014). Placental thrombosis in acute phase abortions during experimental *Toxoplasma gondii* infection in sheep. Vet. Res. 45, 9. doi: 10.1186/1297-9716-45-9 24475786 PMC3931317

[B8] Chaichan P. Mercier A. Galal L. Mahittikorn A. Ariey F. Morand S.. (2017). Geographical distribution of *Toxoplasma gondii* genotypes in Asia: A link with neighboring continents. Infect. Genet. Evol. 53, 227–238. doi: 10.1016/j.meegid.2017.06.002 28583867

[B9] Dard C. Fricker-Hidalgo H. Brenier-Pinchart M. P. Pelloux H. (2016). Relevance of and new developments in serology for toxoplasmosis. Trends Parasitol. 32, 492–506. doi: 10.1016/j.pt.2016.04.001 27167666

[B10] Dubey J. P. Vianna M. C. B. Sousa S. Canada N. Meireles S. Costa J. M. C.. (2006). Characterization of *Toxoplasma gondii* isolates in free-range chickens from Portugal. para 92, 184–186. doi: 10.1645/GE-652R.1 16629334

[B11] Fernández-Escobar M. Calero-Bernal R. Benavides J. Regidor-Cerrillo J. Guerrero-Molina M. C. Gutiérrez-Expósito D.. (2020). Isolation and genetic characterization of *Toxoplasma gondii* in Spanish sheep flocks. Parasit Vectors 13, 396. doi: 10.1186/s13071-020-04275-z 32758283 PMC7404076

[B12] Fernández-Escobar M. Schares G. Maksimov P. Joeres M. Ortega-Mora L. M. Calero-Bernal R. (2022). *Toxoplasma gondii* genotyping: A closer look into Europe. Front. Cell Infect. Microbiol. 12. doi: 10.3389/fcimb.2022.842595 PMC8984497 35402301

[B13] Galal L. Ajzenberg D. Hamidović A. Durieux M.-F. Dardé M.-L. Mercier A. (2018). *Toxoplasma* and Africa: one parasite, two opposite population structures. Trends Parasitol. 34, 140–154. doi: 10.1016/j.pt.2017.10.010 29174610

[B14] Hamilton C. M. Black L. Oliveira S. Burrells A. Bartley P. M. Melo R. P. B.. (2019). Comparative virulence of Caribbean, Brazilian and European isolates of *Toxoplasma gondii* . Parasit Vectors 12, 104. doi: 10.1186/s13071-019-3372-4 30871587 PMC6416883

[B15] Joeres M. Maksimov P. Höper D. Calvelage S. Calero-Bernal R. Fernández-Escobar M.. (2024). Genotyping of European *Toxoplasma gondii* strains by a new high-resolution next-generation sequencing-based method. Eur. J. Clin. Microbiol. Infect. Dis. 43, 355–371. doi: 10.1007/s10096-023-04721-7 38099986 PMC10822014

[B16] Khan A. Dubey J. P. Su C. Ajioka J. W. Rosenthal B. M. Sibley L. D. (2011). Genetic analyses of atypical *Toxoplasma gondii* strains reveal a fourth clonal lineage in North America. Int. J. Parasitol. 41, 645–655. doi: 10.1016/j.ijpara.2011.01.005 21320505 PMC3081397

[B17] Kong J. Grigg M. E. Uyetake L. Parmley S. Boothroyd J. C. (2003). Serotyping of *Toxoplasma gondii* infections in Humans Using Synthetic Peptides. J. Infect. Dis. 187, 1484–1495. doi: 10.1086/374647 12717631

[B18] Largo-de la Torre A. Diezma-Díaz C. Calero-Bernal R. Atencia-Cibreiro G. Sánchez-Sánchez R. Ferre I.. (2022). Archetypal type II and III *Toxoplasma gondii* oocysts induce different immune responses and clinical outcomes in experimentally infected piglets. Front. Immunol. 13. doi: 10.3389/fimmu.2022.1021556 PMC9631316 36341449

[B19] López-Ureña N. M. Calero-Bernal R. González-Fernández N. Blaga R. Koudela B. Ortega-Mora L. M.. (2023). Optimization of the most widely used serological tests for a harmonized diagnosis of *Toxoplasma gondii* infection in domestic pigs. Veterinary Parasitol. 322, 110024. doi: 10.1016/j.vetpar.2023.110024 37729831

[B20] Lorenzi H. Khan A. Behnke M. S. Namasivayam S. Swapna L. S. Hadjithomas M.. (2016). Local admixture of amplified and diversified secreted pathogenesis determinants shapes mosaic *Toxoplasma gondii* genomes. Nat. Commun. 7. doi: 10.1038/ncomms10147 PMC4729833 26738725

[B21] Maksimov P. Basso W. Zerweck J. Schutkowski M. Reimer U. Maksimov A.. (2018). Analysis of *Toxoplasma gondii* clonal type-specific antibody reactions in experimentally infected Turkeys and chickens. Int. J. Parasitol. 48, 845–856. doi: 10.1016/j.ijpara.2018.04.004 29969590

[B22] Maksimov P. Zerweck J. Dubey J. P. Pantchev N. Frey C. F. Maksimov A.. (2013). Serotyping of *Toxoplasma gondii* in cats (Felis domesticus) reveals predominance of type II infections in Germany. PloS One 8, 1–16. doi: 10.1371/journal.pone.0080213 PMC3820565 24244652

[B23] Maksimov P. Zerweck J. Maksimov A. Hotop A. Groß U. Pleyer U.. (2012a). Peptide microarray analysis of in silico -predicted epitopes for serological diagnosis of *Toxoplasma gondii* infection in humans. Clin. Vaccine Immunol. 19, 865–874. doi: 10.1128/CVI.00119-12 22496494 PMC3370440

[B24] Maksimov P. Zerweck J. Maksimov A. Hotop A. Groß U. Spekker K.. (2012b). Analysis of clonal type-specific antibody reactions in *Toxoplasma gondii* seropositive humans from Germany by peptide-microarray. PloS One 7, 1–10. doi: 10.1371/journal.pone.0034212 PMC3314601 22470537

[B25] McLeod R. Boyer K. M. Lee D. Mui E. Wroblewski K. Karrison T.. (2012). Prematurity and severity are associated with toxoplasma gondii alleles (NCCCTS 1981-2009). Clin. Infect. Dis. 54, 1595–1605. doi: 10.1093/cid/cis258 22499837 PMC3348955

[B26] Mukhopadhyay D. Arranz-Solís D. Saeij J. P. J. (2020). Influence of the host and parasite strain on the immune response during *Toxoplasma* infection. Front. Cell Infect. Microbiol. 10. doi: 10.3389/fcimb.2020.580425 PMC7593385 33178630

[B27] Peyron F. Lobry J. R. Musset K. Ferrandiz J. Gomez-Marin J. E. Petersen E.. (2006). Serotyping of *Toxoplasma gondii* in chronically infected pregnant women: predominance of type II in Europe and types I and III in Colombia (South America). Microbes Infection 8, 2333–2340. doi: 10.1016/j.micinf.2006.03.023 16938480

[B28] Sánchez-Sánchez R. Ferre I. Regidor-Cerrillo J. Gutiérrez-Expósito D. Ferrer L. M. Arteche-Villasol N.. (2019). Virulence in mice of a *Toxoplasma gondii* type II isolate does not correlate with the outcome of experimental infection in pregnant sheep. Front. Cell. Infect. Microbiol. 8. doi: 10.3389/fcimb.2018.00436 PMC6328472 30662874

[B29] Sánchez-Sánchez R. Imhof D. Hecker Y. P. Ferre I. Re M. Moreno-Gonzalo J.. (2023). An early treatment with BKI-1748 exhibits full protection against abortion and congenital infection in sheep experimentally infected with *Toxoplasma gondii* . J. Infect. Dis. 229 (2), 558–566. doi: 10.1093/infdis/jiad470 PMC10873186 37889572

[B30] Shwab E. K. Jiang T. Pena H. F. J. Gennari S. M. Dubey J. P. Su C. (2016). The ROP18 and ROP5 gene allele types are highly predictive of virulence in mice across globally distributed strains of *Toxoplasma gondii* . Int. J. Parasitol. 46, 141–146. doi: 10.1016/j.ijpara.2015.10.005 26699401

[B31] Sousa S. Ajzenberg D. Canada N. Freire L. da J. M. C. Dardé M. L.. (2006). Biologic and molecular characterization of *Toxoplasma gondii* isolates from pigs from Portugal. Veterinary Parasitol. 135, 133–136. doi: 10.1016/j.vetpar.2005.08.012 16188390

[B32] Sousa S. Ajzenberg D. Marle M. Aubert D. Villena I. Da Costa J. C.. (2009). Selection of polymorphic peptides from GRA6 and GRA7 sequences of *Toxoplasma gondii* strains to be used in serotyping. Clin. Vaccine Immunol. 16, 1158–1169. doi: 10.1128/CVI.00092-09 19494084 PMC2725539

[B33] Sousa S. Ajzenberg D. Vilanova M. Costa J. Darde M. L. (2008). Use of GRA6-derived synthetic polymorphic peptides in an immunoenzymatic assay to serotype *Toxoplasma gondii* in human serum samples collected from three continents. Clin. Vaccine immunology: CVI 15, 1380–1386. doi: 10.1128/CVI.00186-08 18667636 PMC2546681

[B34] Sousa S. Canada N. Correia da Costa J. M. Dardé M.-L. L. (2010). Serotyping of naturally Toxoplasma gondii infected meat-producing animals. Veterinary Parasitol. 169, 24–28. doi: 10.1016/j.vetpar.2009.12.025 20083355

[B35] Sousa S. Fernandes M. Correia da Costa J. M. (2023). Serotyping, a challenging approach for *Toxoplasma gondii* typing. Front. Med. (Lausanne) 10. doi: 10.3389/fmed.2023.1111509 PMC10115974 37089607

[B36] Stelzer S. Basso W. Benavides Silván J. Ortega-Mora L. M. Maksimov P. Gethmann J.. (2019). *Toxoplasma gondii* infection and toxoplasmosis in farm animals: Risk factors and economic impact. Food Waterborne Parasitol. 15, e00037. doi: 10.1016/j.fawpar.2019.e00037 32095611 PMC7033994

[B37] Su C. Dubey J. P. Ajzenberg D. Khan A. Ajioka J. W. Rosenthal B. M.. (2012). Globally diverse *Toxoplasma gondii* isolates comprise six major clades originating from a small number of distinct ancestral lineages. Proc. Natl. Acad. Sci. 109, 5844–5849. doi: 10.1073/pnas.1203190109 22431627 PMC3326454

[B38] Vallejo R. Benavides J. Arteche-Villasol N. Sánchez-Sánchez R. Calero-Bernal R. Ferreras M. C.. (2023). Experimental infection of sheep at mid-pregnancy with archetypal type II and type III *Toxoplasma gondii* isolates exhibited different phenotypic traits. Vet. Parasitol. 315, 109889. doi: 10.1016/j.vetpar.2023.109889 36753878

